# Salbutamol for analgesia in renal colic: a prospective, randomised, placebo-controlled phase II trial

**DOI:** 10.1136/emermed-2024-214326

**Published:** 2025-04-11

**Authors:** Graham D Johnson, Andrew Tabner, Apostolos Fakis, Rachelle Sherman, Victoria Chester, Andrew Skeggs, Fran Game, Richard Jackson, Suzanne M Mason

**Affiliations:** 1 Emergency Department, University Hospitals of Derby and Burton NHS Foundation Trust, Derby, UK; 2 School of Medicine, University of Nottingham, Nottingham, UK; 3 Clinical Trials Support Unit, University Hospitals of Derby and Burton NHS Foundation Trust, Derby, UK; 4 Health Data Science, University of Liverpool Faculty of Health and Life Sciences, Liverpool, UK; 5 School of Medicine and Population Health, The University of Sheffield, Sheffield, UK

**Keywords:** emergency department, analgesia, Randomized Controlled Trial, urogenital system

## Abstract

**Background:**

The pain of renal colic, mediated in part by ureteral spasm and inflammation, is often severe and difficult to control. Salbutamol has been shown to cause ureteral relaxation, but its effects on the pain of renal colic have never been studied. The objective of this trial was to investigate whether the use of intravenous salbutamol in addition to standard analgesia was associated with greater pain reduction compared with standard analgesia alone in patients presenting to emergency departments (EDs) with renal colic.

**Methods:**

This single-centre, double-blind, phase II, randomised, placebo-controlled trial recruited adult (≥18 years) ED patients with clinically suspected renal colic. Participants were randomised in a 1:1 ratio to receive either 250 µg of intravenous salbutamol or a placebo (0.9% sodium chloride). The primary outcome was the difference in the change in pain scores (measured on a 100 mm Visual Analogue Scale) from baseline to 30 min following trial treatment administration in participants with subsequently confirmed renal colic. A modified intention-to-treat analysis was undertaken for the primary population of participants with confirmed renal colic.

**Results:**

Consent was obtained from 151 patients; 108 participants with confirmed renal colic were included in the primary outcome analysis. There was no statistical difference between groups in median change in pain score at 30 min (salbutamol group −18 mm (IQR −25 to −3), placebo group −13 mm (IQR −33 to −1), difference 5 mm (95% CI −16 to 6, p=0.575)). No significant differences were found in the secondary outcomes related to pain, patient satisfaction or opiate requirement.

More adverse events (AEs) were observed in the salbutamol group (65) compared with placebo (42, p=0.02); no unexpected AEs were identified.

**Conclusions:**

This trial has not identified a clinically or statistically significant benefit from the addition of intravenous salbutamol to standard care for patients presenting to an ED with pain caused by renal colic.

**Trial registration numbers:**

The trial was registered with the European Union Drug Regulating Authorities Clinical Trials Database (EudraCT), reference 2018-004305-11. It was also registered with the ISRCTN Registry, reference 14552440.

**WHAT IS ALREADY KNOWN ON THIS TOPIC**

- The pain of renal colic is severe and difficult to control. Current analgesic regimes are often only partly effective.
- Salbutamol is a beta-adrenoreceptor agonist known to reduce ureteral motility; it has been hypothesised that this may reduce pain in renal colic.

**WHAT THIS STUDY ADDS**

- The addition of salbutamol to usual care for emergency department (ED) patients with either suspected or confirmed renal colic does not result in a clinically significant improvement in their pain.

**HOW THIS STUDY MIGHT AFFECT RESEARCH, PRACTICE OR POLICY**

- There is no scope for further research concerning salbutamol for this indication in an ED setting. Further improvements in the analgesic regime for renal colic are still required.

## Introduction

 Renal colic is the pain experienced when a renal calculus causes partial or complete obstruction of part of the renal outflow tract. The pain arises from ureteric spasm and increased peristalsis, increased pressure in the renal pelvis and prostaglandin release with inflammation.^1^ The lifetime incidence is approximately 12% in men and 6% in women,^2^ with recurrence rates approaching 50%.^3^

Salbutamol is a beta-adrenoreceptor agonist used for multiple indications, with several routes of administration.^4^ It has been hypothesised that beta-adrenoreceptor agonists may reduce the pain of renal colic^5^,^7^ by promoting ureteral relaxation,^8 9^ reducing ureteral contraction frequency^10^ and renal pelvic pressure.^11^ Approximately 60% of an intravenous dose of salbutamol is excreted in the urine^12^; there is therefore potential for systemic and local action.

The standard analgesic regimens for renal colic are often ineffective, and associated with significant side effects. In some studies, fewer than half the participants achieve complete pain relief, many require rescue analgesia within 4 hours,^13^ and drowsiness, nausea and vomiting are frequently reported.^14^

Salbutamol has several potential benefits when compared with existing analgesics. Onset is faster,^14 15^ it can be administered parenterally (avoiding the problems of gastroparesis, nausea and vomiting that are frequently associated with renal colic), and its side effects are generally minor and well tolerated.^4^ Patients with recurrent renal colic may be able to self-medicate with an inhaler, avoiding the need for hospital attendance.

Despite the evidence supporting this potential role for salbutamol in the management of the pain associated with renal colic, there have been no trials to date. This trial aimed to establish whether salbutamol may be an efficacious adjunct when added to the standard analgesic regimen in patients presenting to an emergency department (ED) with confirmed renal colic. Feasibility outcomes were included to inform subsequent phase III trial design.

## Methods

### Study design and setting

This was a single-centre, prospective, randomised, placebo-controlled phase II trial, recruiting in the ED of the Royal Derby Hospital, UK.

### Selection of participants

Potential participants were identified by clinical staff, and eligible individuals were approached, screened, consented and recruited by trial-trained clinicians. Randomisation and preparation of blinded trial medication were performed by trial-trained nursing staff working in an area of the department remote to the participant and were not involved in providing their clinical care. Trial medication was then presented in a syringe (labelled ‘trial medication’) to the clinical staff caring for the participant.

Potential participants were eligible for inclusion if they met the following criteria: capable of giving informed consent; age≥18 years; working diagnosis of renal colic (as determined by the treating clinician); and severe pain requiring intravenous analgesia, and ongoing pain at the time of consent.

A confirmed diagnosis of renal colic was not required for enrolment; this would have introduced a delay while investigations were undertaken.

**Figure 1:**
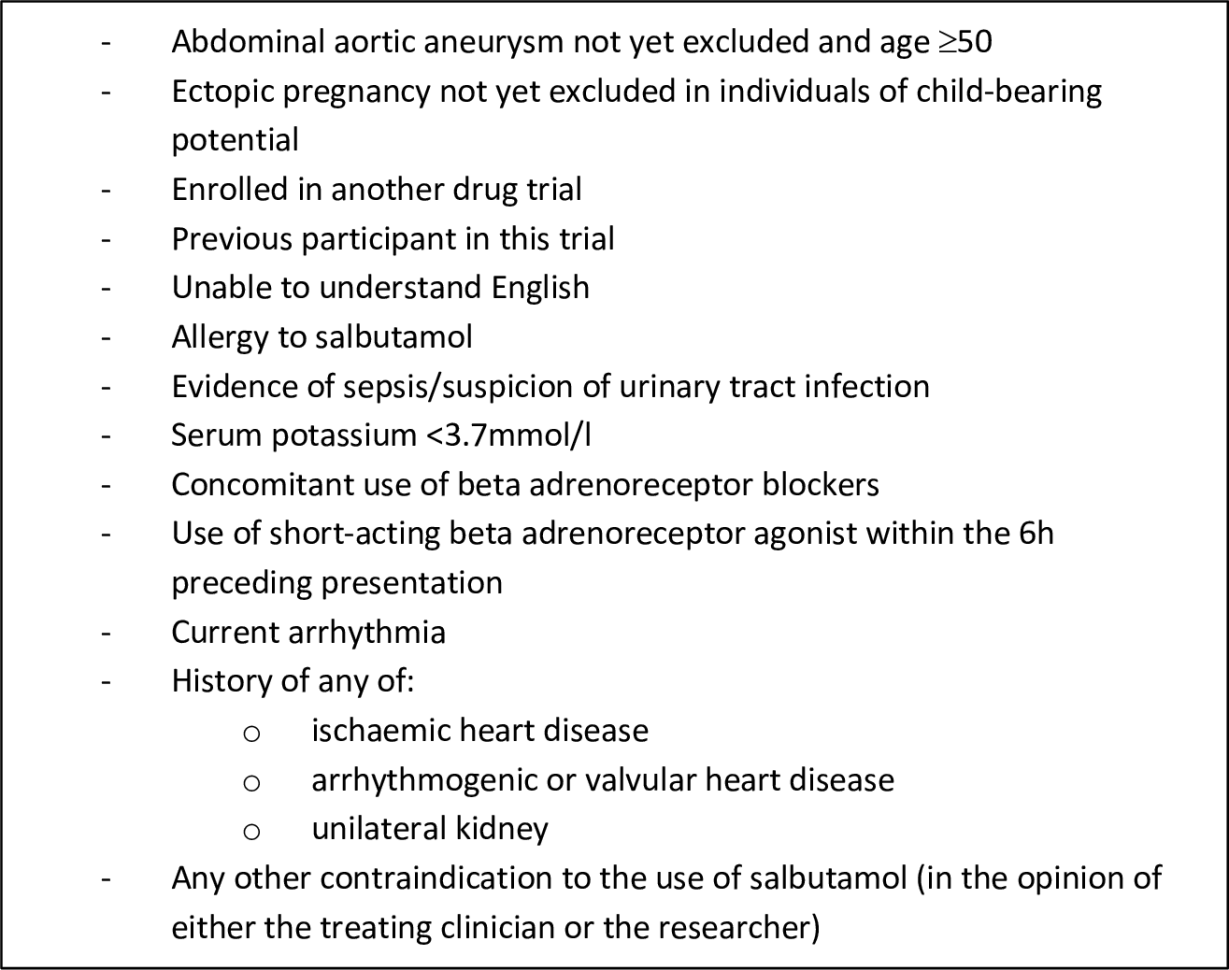
Exclusion criteria.

Exclusion criteria can be seen in figure 1.

### Randomisation

Participants were randomised, with an allocation ratio of 1:1 with no stratification, to one of two groups. A scratch card approach to randomisation was employed^16 17^; this is reported in a separate manuscript.

Randomisation was based on a computer-generated randomisation list created using random permuted blocks of randomly varying size, prepared using NQuery Advisor software by an unblinded statistician and provided to the unblinded site pharmacists.

### Interventions

The intervention group received salbutamol 250 µg, made up to 5 mL with 0.9% sodium chloride, presented as a clear, colourless solution and administered as a slow intravenous bolus over 3–5 min. The control group received 0.9% sodium chloride, presented as a clear, colourless solution and administered as a 5 mL slow intravenous bolus over 3–5 min.

Both groups also received the local usual care analgesic regimen at the treating clinician’s discretion, with no restrictions or stipulations about timing, composition or dosing. This usually included paracetamol (oral or intravenous), a non-steroidal anti-inflammatory (oral ibuprofen or rectal diclofenac) and an opioid (usually intravenous morphine).

### Blinding

Trial participants, treating clinicians, research nurses/practitioners collecting data, the trial statistician performing the analyses, the trial management group (TMG) and the trial steering committee (TSC) were blind to participant allocation.

### Measurements

Baseline demographic data and physiological variables were recorded. Participants were supported to complete a case report form (CRF) for the first 2 hours of their trial involvement, with measurements taken at baseline and then at 15, 30, 60 and 120 min following investigational medicinal product (IMP) administration. These included pain score on a 100 mm Visual Analogue Scale (VAS), McGill Pain Questionnaire^18^ completion and any adverse effects. Participants independently completed a CRF concerning the remainder of their trial involvement at 4, 8, 12, 16, 20 and 24 hours after IMP administration.

### Primary outcome

The trial primary outcome was the difference between the salbutamol and placebo arms in the change in pain scores from baseline to 30 min following IMP administration in participants with confirmed renal colic. While speed of onset of action is an important outcome, it was felt that sustained relief was also important and 30 min as a *primary* outcome was a suitable measure.

### Secondary outcomes

The following key secondary outcomes are presented; a full list can be found in online supplemental material 1:

1. The difference in the change from baseline pain score to pain scores at the following time points between trial arms: 15 min, 30 min, 60 min, 120 min, 240 min and then 4 hourly thereafter, until 24 hours post drug administration or hospital discharge (whichever happens first) in participants with both confirmed and suspected renal colic (see below Prespecified group definitions).
2. The difference in the change in qualitative pain description from baseline pain assessment to pain assessments at the following time points between trial arms as measured using the short-form McGill Pain Questionnaire: 15 min, 30 min, 60 min, 120 min post drug administration.
3. Frequency and dose of morphine during the first 24 hours from enrolment.
4. Frequency of development of acute kidney injury (AKI) and date of occurrence if present.
5. Presence/absence, site and size of renal calculus.
6. Side effects of trial treatment.
7. Feasibility outcomes to inform subsequent trial design.

### Prespecified group definitions

- Suspected renal colic: the entire trial population at enrolment.

- Confirmed renal colic: a subgroup of the enrolled trial population (but the cohort informing the primary outcome), defined as either:
  - Patients with evidence of a renal calculus on the side of their abdominal/flank pain as proven by imaging during the index presentation, and a discharge diagnosis consistent with renal colic.
  - Patients with a history of a proven renal calculus and an ED diagnosis of renal colic and (where relevant) a hospital discharge diagnosis consistent with renal colic.
  - Patients who pass a renal calculus while in the ED (visually confirmed by either patient or staff member).

### Sample size

This was a phase II trial to demonstrate an efficacy signal on the primary outcome. The sample size estimation was therefore estimated based on the ‘probability of benefit’ approach using the Mann-Whitney U test with R software.^19^

The minimum clinically important difference (MCID) in pain on a VAS in ED patients has previously been described as 13 mm.^20 21^ This has been assumed as the MCID between the salbutamol and placebo arms. Assuming an SD of 20 mm,^13^ then at 5% significance level with 90% power, 106 participants with *confirmed* renal colic were required.

### Statistical methods

The primary outcome was compared between the two trial arms using the Mann-Whitney U test. Secondary analysis of the primary outcome was carried out using an analysis of covariance (ANCOVA) approach, analysing the pain scores at 30 min and including the baseline pain scores, age, gender and weight as covariates.

A ‘modified intention-to-treat’ (ITT) analysis of the primary outcome was carried out within the ‘*confirmed* renal colic’ group on the full data set, by retaining patients in their initially randomised groups irrespective of any protocol violations or the treatment they actually received. Secondary analysis of the primary outcome was also carried out on the ‘per-protocol’ principle by excluding any patients with major protocol deviations and on the ‘as-treated’ principle by including patients in the treatment group of the actual medication they have received. The secondary outcome of the change in pain scores (measured with VAS) from baseline to 30 min in patients with ‘*suspected* renal colic’ was compared between the two groups using the Mann-Whitney U test.

The analysis of the primary outcome was undertaken on the complete cases dataset instead of imputing the baseline and 30 min VAS scores for two participants, as described in the protocol; it was determined that imputation of two values using multiple imputation would not add any value in the statistical analysis or interpretation of results. Furthermore, the required sample of 106 patients with confirmed renal colic and complete VAS scores at 30 min had been reached. Two participants did not have pain at the intended time of trial medication administration and were therefore withdrawn prior to drug administration. Their baseline VAS scores were included in the modified ITT analysis. Their missing VAS pain scores at 30 min were replaced with ‘0’ as no pain had been indicated, instead of performing multiple imputations.

The change in VAS scores and in sensory, affective and total McGill scores from baseline to 15, 60, 120, 240 min and 4 hourly thereafter in patients with ‘confirmed renal colic’ and with ‘suspected renal colic’ was compared between the two trial arms at each time point using the Mann-Whitney U test. Responses in the patient satisfaction questionnaire were compared by treatment group using a Kruskal-Wallis test. Total morphine dose prior and after the trial treatment was compared between the two treatment groups using the Mann-Whitney U test. The presence of AKI within 7 days from admission was compared between the two treatment groups using the χ^2^ test. The treatment that the patients think they received was compared with the actual treatment they received using the χ^2^ test.

#### Toxicity

The number and percentage of patients reporting a Serious Adverse Event (SAE) or Suspected Unexpected Serious Adverse Reaction (SUSAR) were summarised by treatment group and compared using a χ^2^ test. Analysis of adverse events (AEs) was restricted to participants who received the allocated trial medication (‘as-treated’ analysis group), so that absence or occurrence of harm was not attributed to a treatment that was never received.

Results from Mann-Whitney U tests were reported as the difference in medians between the two groups, together with associated 95% CIs using the Bonett-Price method. Results from the ANCOVA were reported as the difference in means between the two groups, standard errors and their associated 95% CIs. Analysis was assessed using two-sided 0.05 level. Stata statistical software was used for the analysis. Details of further statistical analyses can be found in online supplemental file 1.

### Patient and public involvement (PPI)

PPI group members assisted with research question development, grant application, study design (including frequency of observations and burden of interventions) and writing participant-facing documents. They served on the TMG and TSC.

### Registration details

The trial was registered with the European Union Drug Regulating Authorities Clinical Trials Database (EudraCT), reference 2018-004305-11. It was also registered with the ISRCTN Registry, reference 14552440.

## Results

Recruitment took place between 17 September 2019 and 14 March 2020, paused due to the COVID-19 pandemic, restarted on 17 November 2020 and finished on 20 September 2022.

### Participant flow

**Figure 2:**
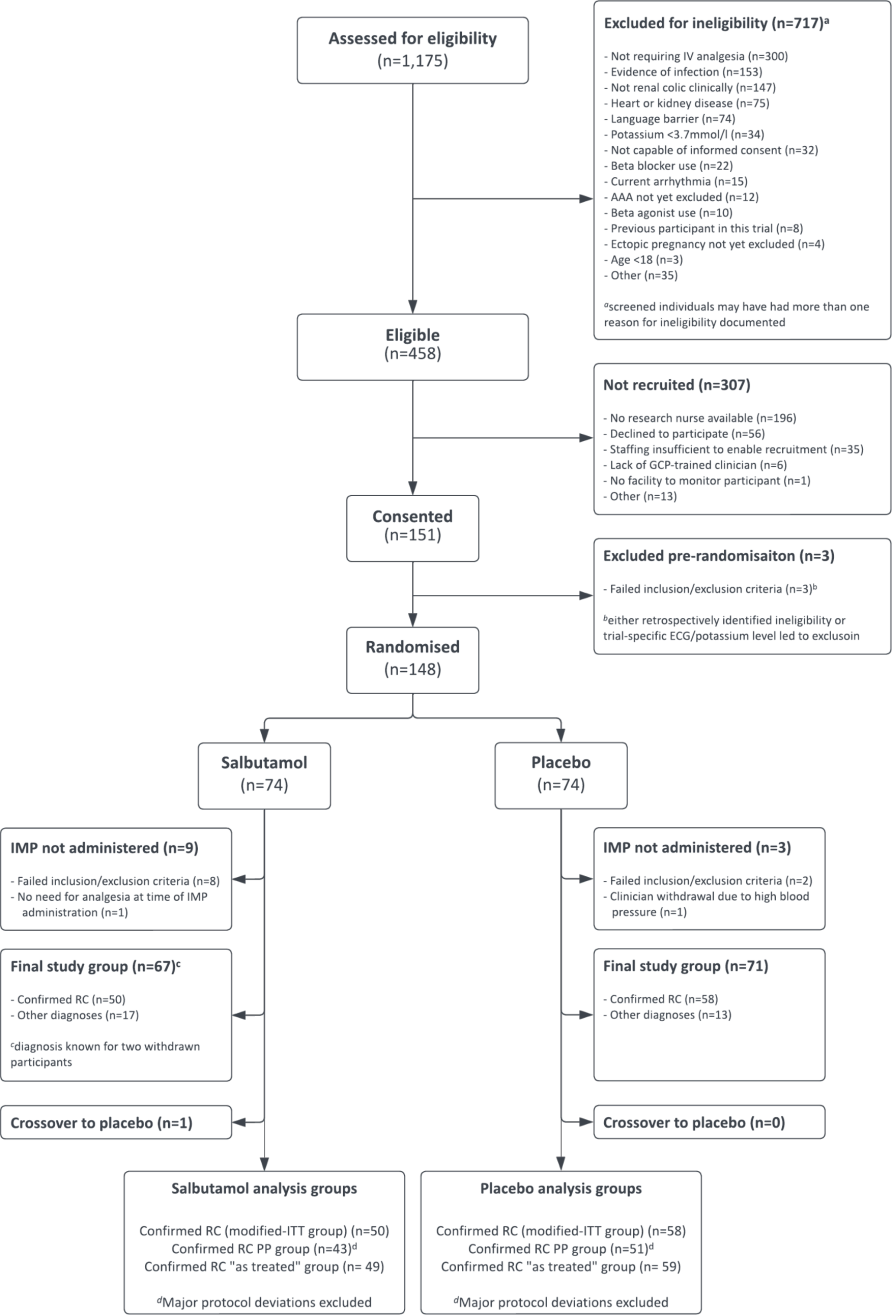
Consolidated Standards of Reporting Trials (CONSORT) diagram demonstrating flow of participants through the trial. ITT, intention-to-treat; PP, per-protocol; RC, renal colic; IV, intravenous; AAA, abdominal aortic aneurysm; GCP, Good Clinical Practice.

A total of 1175 patients were assessed for eligibility; of these, 458 were eligible and 151 provided informed consent (figure 2). Three were subsequently excluded due to ECG findings or potassium level, and 148 participants with suspected renal colic were therefore randomised (74 in each group). In the salbutamol group, 9 participants did not complete the study, but the final diagnosis group was known for 67, including 2 withdrawn participants for whom imaging results (and therefore final diagnosis) were available prior to participant randomisation, but whose pain resolved prior to trial treatment. In the placebo group, 3 patients did not complete the study, and the final diagnosis group was known for 71 participants.

### Characteristics of study subjects

Demographic and baseline clinical characteristics can be found in table 1. Groups were comparable at randomisation, both in terms of demographics and baseline pain scores.

**Table 1:** Baseline demographic data and clinical variables for all participants

Baseline data	Salbutamol (n=74)	Placebo (n=74)
Age (years) mean (SD)	42 (12)	46 (14)
Weight (kg) mean (SD)	89 (23)	93 (22)
Sex (male) n (%)	50 (68)	52 (70)
Baseline pain score (mm) median (IQR)	(n=68) 67 (49–83)	(n=72) 67 (50–86)
Presence of stone (yes) n (%)	(n=66) 47 (71)	(n=70) 55 (79)
Stone size (mm) mean (SD)	(n=43) 4 (1)	(n=52) 5 (2)
Site of stone n (%)	(n=45)	(n=55)
Proximal third	11 (24)	21 (38)
Middle third	3 (7)	7 (13)
Distal third	31 (69)	27 (49)
Baseline pain score (confirmed renal colic) (mm) median (IQR**)**	(n=49) 67 (48–82)	(n=58) 67 (49–89)

There were no significant differences in baseline participant characteristics or study outcomes when analysed by pre-COVID-19 or post-COVID-19 recruitment periods; this analysis was not prespecified, but was recommended by the National Institute for Health Research for studies where recruitment was interrupted by the COVID-19 pandemic.

### Primary outcome data

**Figure 3:**
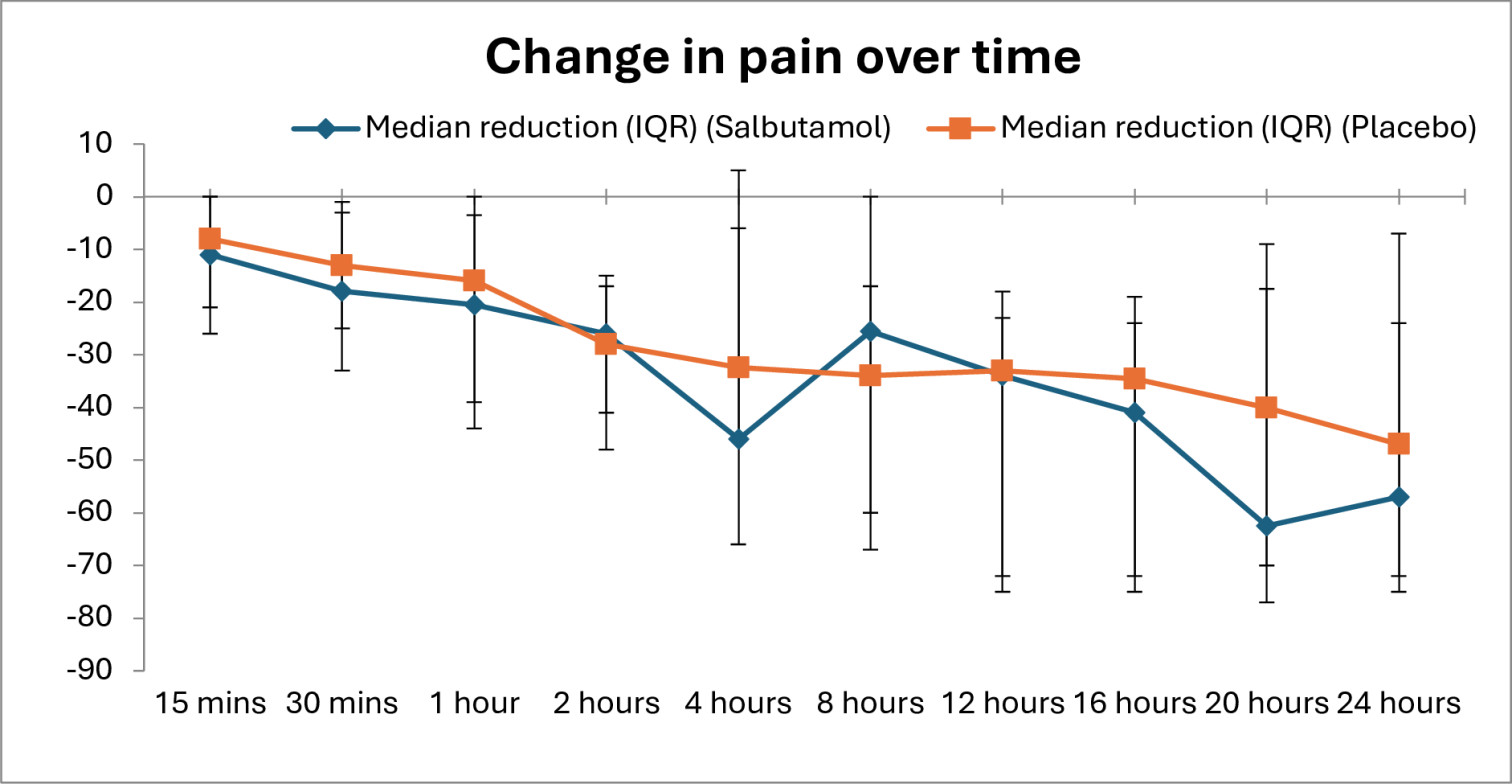
Change in pain score from baseline (100 mm Visual Analogue Scale) at all study time points in participants with confirmed renal colic analysed on an intention-to-treat basis.

The median change in pain score for participants with *confirmed* renal colic in the salbutamol group at 30 min when compared with baseline was −18 mm (IQR −25 to −3) and in the placebo group −13 mm (IQR −33 to −1); this represents a difference of −5 mm (95% CI −16 to 6, p=0.575) favouring the salbutamol group. This does not meet the prespecified MCID of 13 mm, nor was it statistically significant. Pain scores at all time points can be seen in figure 3.

Sensitivity analyses conducted on the primary outcome on both ‘per-protocol’ and ‘as-treated’ bases did not change either the clinical or statistical significance of the primary outcome (table 2).

**Table 2:** Change in pain scores on 100mm Visual Analogue Scale (mm) at 30 min, compared with baseline, in participants with confirmed renal colic

Analysis type	Salbutamol median (IQR**)**	Placebo median (IQR**)**	Difference in medians (95% CI**)**	P value
Intention-to-treat	(n=49)−18 (−25 to −3)	(n=57)−13 (−33 to −1)	−5 (−16 to 6)	0.575
Per-protocol	(n=42)−18 (−27 to −3)	(n=51)−13 (−34 to −1)	−5 (−15 to 5)	0.309
As-treated	(n=48)−18 (−26 to −3)	(n=58)−11.5 (−33 to −1)	−6.5 (−17 to 4)	0.439

The secondary ANCOVA analysis of the primary outcome, including the baseline pain scores, age, gender and weight as covariates confirmed the results of the primary analysis (table 3).

**Table 3:** Secondary ANCOVA analysis of the primary outcome

Secondary analysisConfirmed renal colic	Parameter estimate(95% CI**)**	SE	P value*
Difference between treatment groups in change in pain score (VAS mm) from baseline to 30 min†Treatment (salbutamol compared with placebo)	(n=101)−4 (−13 to 5)	4.7	0.369
Difference between treatment groups in change in pain score (VAS mm) from baseline to 30 min‡Treatment (salbutamol compared with placebo)	(n=95)−4.5 (−14 to 5)	4.9	0.354

* Based on ANCOVA including baseline pain score, age, gender and weight as covariates.

† If weight at screening was missing, the recorded value in patient’s records within 12 months from consent was used.

‡ If weight at screening was missing, then this patient record was excluded.

VAS, Visual Analogue Scale.

### Secondary outcome data

#### Pain

No clinically or statistically significant difference in the change in pain scores from baseline to 30 min between groups was observed for participants with *suspected* renal colic, analysed on an ITT basis (−5 mm (95% CI −14 to 4); p=0.281).

No clinically or statistically significant differences in the change in pain scores from baseline were observed between groups for participants with either confirmed or suspected renal colic, analysed on an ITT basis, at any time point during the study (table 4).

**Table 4:** Change in pain score from baseline at all study time points, together with the difference in change in pain scores, for both the confirmed and suspected renal colic groups

Time after IMP	Change in pain score on 100 mm Visual Analogue Scale							
	Confirmed renal colic (n=106**)**				Suspected renal colic (n=137**)**			
	Salbutamol median (IQR**)**	Placebo median (IQR**)**	Difference in medians (95% CI**)**	P value	Salbutamol median (IQR**)**	Placebo median (IQR**)**	Difference in medians (95% CI**)**	P value
15 min	(n=48)−11 (−26 to 0)	(n=58)−8 (−21 to 0)	−3 (−10 to 4)	0.365	(n=65)−13 (−23 to 0)	(n=71)−8 (−21 to 0)	−5 (−11 to 1)	0.299
30 min	(n=49)−18 (−25 to −3)	(n=57)−13 (−33 to −1)	−5 (−16 to 6)	0.575	(n=66)−18 (−33 to −6)	(n=71)−13 (−33 to −1)	−5 (−14 to 4)	0.281
1 hour	(n=48)−20.5 (−39 to −3.5)	(n=58)−16 (−44 to 0)	−4.5 (−19 to 10)	0.673	(n=64)−20 (−39 to −3.5)	(n=71)−18 (−41 to 0)	−2 (−12 to 8)	0.693
2 hours	(n=47)−26 (−48 to −15)	(n=57)−28 (−41 to −17)	2 (−11 to 15)	0.965	(n=63)−25 (−48 to −7)	(n=69)−28 (−41 to −16)	3 (−7 to 13)	0.731
4 hours	(n=19)−46 (−66 to −6)	(n=24)−32.5 (−46 to 5)	−13.5 (−45 to 18)	0.199	(n=22)−42.5 (−56 to −6)	(n=28)−23 (−44 to 4)	−19.5 (−45 to 6)	0.140
8 hours	(n=10)−25.5 (−67 to 0)	(n=21)−34 (−60 to −17)	8.5 (−39 to 56)	0.582	(n=11)−20 (−67 to 0)	(n=23)−32 (−60 to −16)	12 (−30 to 52)	0.519
12 hours	(n=9)−34 (−75 to −23)	(n=16)−33 (−72 to −18)	−1 (−54 to 52)	0.988	(n=10)−31.5 (−75 to −15)	(n=19)−32 (−82 to −7)	0.5 (−45 to 46)	0.883
16 hours	(n=9)−41 (−75 to −24)	(n=14)−34.5 (−72 to −19)	−6.5 (−54 to 41)	0.890	(n=10)−39 (−75 to 4)	(n=17)−34 (−64 to −19)	−5 (−48 to 38)	0.912
20 hours	(n=8)−62.5 (−70 to −17.5)	(n=15)−40 (−77 to −9)	−22.5 (−67 to 22)	1.000	(n=9)−61 (−69 to −12)	(n=17)−40 (−74 to −9)	−21 (−68 to 26)	0.905
24 hours	(n=7)−57 (−75 to −7)	(n=16)−47 (−72 to −24)	−10 (−54 to 34)	0.962	(n=9)−57 (−62 to 8)	(n=18)−47 (−68 to −23)	−10 (−55 to 35)	0.519

P value based on Mann-Whitney U test.

IMP, Investigational Medicinal Product.

#### McGill Pain Questionnaire

No statistically significant differences between groups were identified in any component of the McGill Pain Questionnaire at any time point in the confirmed renal colic group. Salbutamol did not demonstrate any analgesic-sparing effects in participants with confirmed renal colic.

The regression analysis for secondary pain outcomes can be found in online supplemental material 3; no significant results were identified.

#### Satisfaction questionnaire

There was no significant difference between groups in the level of agreement with the statement “my pain has been well controlled” (p=0.174), with the majority of participants in both groups (over 90%) either agreeing or strongly agreeing with the statement.

#### Morphine requirement

There was no significant difference between groups in morphine dose received either prior to trial enrolment or after IMP administration (online supplemental material 4).

#### Other secondary outcomes

Two participants in the placebo group had AKI on presentation; two patients in the salbutamol group and one patient in the placebo group developed AKI within 7 days of recruitment (p=0.837).

There were no statistically significant differences in any of the clinical secondary outcomes.

#### Side effects and AEs

An increase in pulse rate from baseline in the salbutamol group compared with the placebo group was observed at 15, 30, 60 and 120 min (online supplemental material table 5a).

There were 65 AEs reported by 34 participants (69%) in the salbutamol group, while in the placebo group 42 AEs were reported by 28 participants (47%, p=0.022). The most common reported AEs in the salbutamol group were tremor (23 (35%)), palpitations (13 (20%)) and dizziness (7 (11%)). In the placebo group, the most common reported AEs were headache (13 (33%)), dizziness (5 (12%)), nausea (5 (12%)) and palpitations (5 (12%)). There was no statistical difference between groups in level of agreement with the statement “the side effects of the painkillers were minimal” in the satisfaction questionnaire (p=0.181) (online supplemental material 5b).

#### Feasibility

The trial recruited to time and target, recruiting approximately one-third of the eligible patients attending the ED; this is consistent with the recruitment rate noted in other ED studies.^22^

Participants were asked whether they knew which arm of the study they had been randomised to; 49% of participants in the salbutamol arm correctly guessed their allocation, compared with 20% of those in the placebo arm (p=0.024).

## Discussion

This pragmatic phase II trial compared salbutamol with placebo as an analgesic adjunct in the management of renal colic in the ED. The primary population of interest used in analyses of the primary endpoint comprised patients with *confirmed* renal colic. However, this diagnosis was frequently not confirmed at the time of randomisation; the whole trial population was therefore participants with *suspected* renal colic. The ITT principle was applied in the patients with *confirmed* renal colic, and hence the term ‘modified ITT’ was used.

The salbutamol group had lower pain scores between 15 and 60 min, but this difference did not reach prespecified thresholds for clinical significance. Similarly, there was no significant difference between groups with respect to the McGill Pain Questionnaire or other surrogate markers of pain, including other analgesic use, satisfaction with pain relief or length of hospital stay.

Pragmatic clinical trials^23^ investigate the effectiveness of an intervention in the clinical setting and patient population in whom it is proposed for use, and the ED setting, more than most, is fraught with challenges to ‘scientific purity’. Patients are heterogeneous in their underlying diagnoses, the timing of their presentation with respect to symptom onset, the prehospital care received, and their initial ED triage, assessment and management. This can result in a trial treatment being administered at various stages of both the disease process and the treatment pathway. Confounding factors are challenging to control for and render interpretation of results more difficult. Nevertheless, a pragmatic trial is arguably more useful than a trial with prespecified ‘standard’ care and a tightly selected participant group bearing little resemblance to day-to-day work. A strength of this study is that the recruited sample accurately represents the cohort in whom clinicians might consider the use of the trial intervention; the demonstrated lack of efficacy in this context can immediately inform clinical practice.

Beta-adrenoreceptor agonists are not the only class of drug known to cause ureteral relaxation; alpha adrenoreceptor blockers (eg, tamsulosin) have the same effect on ureteral motility and are frequently used in the treatment of renal calculi to reduce time to stone passage.^24^ In some studies, they are also found to reduce the number of pain episodes and other analgesic requirements.^24 25^ This trial was neither designed nor powered to assess the impact of salbutamol on the number of pain episodes or time to stone passage. The duration of action for oral alpha adrenoreceptor blockers far exceeds that of salbutamol and renders them more useful for longer-term management of renal calculi. However, they are not suitable for the management of acute pain due to their pharmacokinetics^26^; absorption of oral medications is also frequently impaired in acute severe pain.^27^

Participants in the salbutamol group reported more adverse reactions than those in the placebo group; no unanticipated AEs were identified. Most participants considered the side effects experienced to be mild; this is notable given that salbutamol was administered intravenously in this study, which is generally associated with a greater frequency and severity of side effects than other routes.^28^

The clinical problem driving the development of this trial was a recognition that current analgesic regimens for renal colic are frequently ineffective, with many patients not achieving adequate pain relief and requiring rescue analgesia.^13^ The median baseline pain score for both trial groups was 67 mm despite participants having received standard analgesics; this is consistent with previous studies. Furthermore, most participants reported ongoing pain throughout the trial period. This trial therefore confirms the need for further research to improve analgesia in renal colic. A variety of research targets are evident; more potent opioids (eg, fentanyl) are used infrequently, but studies have demonstrated impressive analgesic efficacy.^29^ There may also be a role for ketamine in the management of severe renal colic.^30^ Further research concerning their effectiveness (and that of other novel agents) alongside existing analgesic regimens is required prior to their widespread adoption.

### Limitations

Unusually for a phase II trial, the analgesic efficacy of salbutamol as a standalone agent was not assessed. A head-to-head comparison with known analgesics would potentially have deprived participants in the salbutamol group of any (known) effective analgesia and would not have been ethically justifiable. Furthermore, restricting recruitment to participants who had not received any prior analgesia would have rendered recruitment infeasible.

Salbutamol is associated with readily recognisable physiological effects (eg, tremor) that were likely apparent to both treating clinicians and researchers. Participants who received salbutamol were more likely to correctly guess their treatment allocation than those who received placebo. Blinding in this trial was therefore at best only partially effective.

There was frequently an unavoidable delay between participant consent and trial treatment administration. Resources for pre-prepared, blinded trial treatments were not available, and the study processes relied on staff availability to prepare trial medications. Given the speed of onset is a theoretical advantage of salbutamol, this may have reduced its measured efficacy. It may also have led to a change in pain level between identification and baseline measurements (which took place immediately prior to IMP administration).

## Conclusion

This trial has not identified a clinically significant benefit from the addition of intravenous salbutamol to standard care for ED patients with severe pain caused by renal colic. While it cannot be stated that salbutamol has no analgesic effect, further exploration of its role as part of a multimodal analgesic regimen administered in an ED setting is not warranted. Pain control in patients with renal colic has again been demonstrated to be challenging, and further work is required to optimise care in this cohort.

## Supplementary material

10.1136/emermed-2024-214326 online supplemental file 1

## Data Availability

Data are available on reasonable request.
