# Supplementary material for: Salbutamol for analgesia in renal colic: a prospective, randomised, placebo-controlled phase II trial
Source: Emerg Med J. 2025 Apr 11;42(6):e214326. doi: 10.1136/emermed-2024-214326 (PMC12171521; doi:10.1136/emermed-2024-214326)
Supplement: online supplemental file 1 [file emermed-42-6-s001.pdf]

# Supplemental Material

**Supplemental Material 1 - Secondary clinical and feasibility outcomes**

1. The difference in the change in pain scores (measured on an 100mm Visual Analogue Scale [VAS]) from baseline to 30 minutes post drug administration between trial arms in patients with "**Suspected** Renal Colic".
2. The difference in the change from baseline pain score to pain scores at the following time points between trial arms: 15min, 60min, 120min, 240min, and then four-hourly thereafter, until 24 hours post drug administration or hospital discharge (whichever happens first) in both of the above subgroups.
3. The difference in the change in qualitative pain description from baseline pain assessment to pain assessments at the following time points between trial arms as measured using the short-form McGill Pain Questionnaire: 15min, 30min, 60min, 120min post drug administration.
4. Frequency and dose of morphine during the first 24 hours from enrolment (including prehospitally).
5. Any other analgesics required and the timing of their administration.
6. Length of hospital stay.
7. Presence/absence, site, and size of renal calculus.
8. Frequency of development of Acute Kidney Injury and date of occurrence if present.*
9. Degree of hydronephrosis (if present) as identified on routine imaging.
10. Side effects of trial treatment.
11. The mean and standard deviation of the primary outcome.
12. Feasibility outcomes to inform subsequent trial design, including:
    - Screening rate
    - Randomisation rate
    - Recruitment rate
    - Participant retention
    - Any identified process issues
    - Volume of missing data
    - Patient compliance with trial assessments
    - Proportion of enrolled patients with confirmed renal colic
    - Emergency department diagnosis
    - Hospital discharge diagnosis
    - Patient satisfaction with the trial medication, process and delivery within the ED, including their belief regarding arm of the trial to which they were randomised

*outcome added after trial recruitment commenced at the advice of the DMEC– data for already-recruited participants collected retrospectively.

Data for any secondary outcomes results not presented in the manuscript or supplementary material are available upon reasonable request to the corresponding author

**Supplemental Material 2 – Secondary outcome statistical analysis plan**

The analysis of secondary outcomes, e.g. the VAS scores at latest follow up points (4, 8, 12, 16, 20, and 24 hours), was undertaken on complete case analysis due to very high rate of missing data of around 85%. Results from Chi-Squared / Fisher's Exact test were reported as the difference in proportions and ratio of proportions with their associated 95% confidence intervals.

**Pain Scores**

The change in pain scores (measured with VAS) from baseline to 15, 60, 120, 240 minutes, and four-hourly thereafter in patients with "**Confirmed** Renal Colic" and with "**Suspected** Renal Colic" was compared between the two trial arms across all time points using linear mixed effects regression models including the baseline pain scores, age, gender and weight as covariates.

The change in pain scores (measured with McGill Pain Questionnaire) from baseline to 15, 30, 60, and 120 minutes in patients with "**Confirmed** Renal Colic" and with "**Suspected** Renal Colic" was compared between the two trial arms across all time points using linear mixed effects regression models including the baseline pain scores, age, gender and weight as covariates.

**Clinical Outcomes**

Secondary continuous outcomes (timing of morphine dose, frequency of morphine, and total morphine dose within 6 hours from trial treatment; vital signs, length of stay) were compared between the two treatment groups using Mann U Whitney. Secondary categorical outcomes (morphine required, and other analgesics required prior and after the trial treatment, concomitant medications required, diagnosis, McGill pain categories) were compared between the two treatment groups using Chi-squared test/Fishers Exact test/Kruskal-Wallis test as appropriate.

**Feasibility Outcomes**

Descriptive statistics were presented to summarise the feasibility outcomes across each of the randomisation groups, where relevant. The continuous feasibility outcomes were reported with medians & Interquartile Ranges (IQR), while the categorical feasibility outcomes will be reported with frequencies & percentages.

**Supplemental Material 3 – Regression analyses of secondary outcomes**

| Confirmed renal colic | Parameter Estimate (n=19) (95% cis) | p-value* |
|---|---|---|
| **Change in pain score (VAS) from baseline to follow up timepoints** (Ref: *Placebo @15mins*) | | |
| Salbutamol@30mins | -4 (-34 to 27) | 0.820 |
| Salbutamol@60mins | -18 (-49 to 13) | 0.254 |
| Salbutamol@120mins | 8 (-22 to 39) | 0.594 |
| Salbutamol@4hours | 18 (-13 to 48) | 0.260 |
| Salbutamol@8hours | 27 (-4 to 58) | 0.087 |
| Salbutamol@12hours | 16 (-14 to 47) | 0.307 |
| Salbutamol@16hours | 16 (-15 to 46) | 0.317 |
| Salbutamol@20hours | 11 (-20 to 42) | 0.480 |
| Salbutamol@24hours | 12 (-19 to 43) | 0.450 |

*Based on Linear Mixed Models including treatment by time interaction including the baseline pain scores, age, sex, and weight as covariates, and patient ID as a random effect.

| Confirmed renal colic | Parameter Estimate (n=101) (95% cis) | p-value* |
|---|---|---|
| **Change in Sensory Mcgill score from baseline to follow up timepoints** (Ref: Placebo@15mins) | | |
| Salbutamol@30mins | 0.6 (-1.6 to 2.8) | 0.586 |
| Salbutamol@60mins | 1.1 (-1.1 to 3.3) | 0.319 |
| Salbutamol@120mins | 1.9 (-0.2 to 4.1) | 0.082 |
| **Change in Affective Mcgill score from baseline to follow up timepoints (**Ref:Placebo) | | |
| Salbutamol@30mins | 0.2 (-0.7 to 1.1) | 0.670 |
| Salbutamol@60mins | 0.5 (-0.3 to 1.4) | 0.228 |
| Salbutamol@120mins | 0.5 (-0.4 to 1.4) | 0.266 |
| **Change in Total Mcgill score from baseline to follow up timepoints (**Ref:Placebo) | | |
| Salbutamol@30mins | 0.8 | 0.584 |
| Salbutamol@60mins | 1.7 | 0.257 |
| Salbutamol@120mins | 2.4 | 0.095 |

* Based on Linear Mixed Models including treatment by time interaction including the baseline pain scores, age, sex, and weight as covariates, and patient ID as a random effect.

| Suspected renal colic | **Parameter Estimate** (n=22) (95% Cis) | **p-value*** |
|---|---|---|
| **Change in pain score (VAS) from baseline to follow up timepoints** (Ref: *Placebo @15mins*) | | |
| Salbutamol@30mins | -6 (-34 to 22) | 0.683 |
| Salbutamol@60mins | -16(-44 to 12) | 0.264 |
| Salbutamol@120mins | 16 (-12 to 44) | 0.267 |
| Salbutamol@4hours | 17 (-11 to 45) | 0.239 |
| Salbutamol@8hours | 28 (0.4 to 56) | 0.047 |
| Salbutamol@12hours | 16 (-11 to 45) | 0.241 |
| Salbutamol@16hours | 21 (-7 to 49) | 0.147 |
| Salbutamol@20hours | 18 (-10 to 46) | 0.201 |
| Salbutamol@24hours | 19 (-9 to 47) | 0.178 |

*Based on Linear Mixed Models including treatment by time interaction including the baseline pain scores, age, sex, and weight as covariates, and patient ID as a random effect.

| **Suspected renal colic** | **Parameter Estimate** (n=132) (95% cis) | **p-value*** |
|---|---|---|
| **Change in Sensory McGill score from baseline to follow up timepoints** (Ref: Placebo@15mins) | | |
| Salbutamol@30mins | 0.1 (-1.7 to 1.9) | 0.923 |
| Salbutamol@60mins | 0.5 (-1.3 to 2.3) | 0.566 |
| Salbutamol@120mins | 1.9 (0.1 to 3.7) | 0.043 |
| **Change in Affective McGill score from baseline to follow up timepoints** (Ref: Placebo@15mins) | | |
| Salbutamol@30mins | 0.1 (-0.6 to 0.8) | 0.794 |
| Salbutamol@60mins | 0.4 (-0.4 to 1.1) | 0.347 |
| Salbutamol@120mins | 0.4 (-0.3 to 1.2) | 0.235 |
| **Change in Total McGill score from baseline to follow up timepoints** (Ref: Placebo@15mins) | | |
| Salbutamol@30mins | 0.2 (-2.2 to 2.6) | 0.877 |
| Salbutamol@60mins | 0.9 (-1.5 to 3.2) | 0.465 |
| Salbutamol@120mins | 2.3 (-0.1 to 4.7) | 0.056 |

* Based on Linear Mixed Models including treatment by time interaction including the baseline pain scores, age, sex, and weight as covariates, and patient ID as a random effect.

## Supplemental Material 4 - Morphine Requirements

| **Morphine requirement** | **Salbutamol** median (IQR) | **Placebo** median (IQR) | **Difference in medians** (95% CI) | **p-value** |
|---|---|---|---|---|
| **Morphine administered prior to IMP** (mg) | (n=35)<br>10 (10 to 20) | (n=40)<br>10 (10 to 15) | 0 (-3 to 3) | 0.695 |
| **Morphine administered after IMP** (mg) | (n=15)<br>10 (8 to 10) | (n=23)<br>10 (5 to 10) | 0 (-1 to 1) | 0.921 |
| **Morphine administered within 6h after IMP** (mg) | (n=12)<br>10 (9 to 10) | (n=21)<br>10 (5 to 10) | 0 (-2 to 2) | 0.699 |

**Supplemental Material 4.** Morphine requirements

## Supplemental Material 5 - Side effect and Adverse Event Data

| Change in pulse rate compared to baseline | | | | |
|---|---|---|---|---|
| **Time point after administration** | **Confirmed renal colic** (n=106) | | | |
| | **Salbutamol** (bpm) | **Placebo** (bpm) | **Difference in medians** (95% CI) | **p-value** |
| **15 minutes** Median (IQR) | (n=48) 27 (16 to 33) | (n=58) -1 (-5 to 4) | 28 (23 to 33) | <0.0001 |
| **30 minutes** Median (IQR) | (n=47) 20 (10 to 27) | (n=56) -0.5 (-6 to 4) | 20.5 (16 to 25) | <0.0001 |
| **60 minutes** Median (IQR) | (n=48) 12 (4 to 20) | (n=58) -1 (-6 to 5) | 13 (9 to 17) | <0.0001 |
| **120 minutes** Median (IQR) | (n=46) 8 (2 to 15) | (n=56) -1.5 (-4 to 7) | 9.5 (4 to 15) | 0.000 |

**Supplementary Material Table 5a.** Change in pulse rate compared to baseline in the confirmed renal colic group at all statistically significant time points.

| **Level of agreement** n (%) | **Salbutamol n=43** | **Placebo n=50** | **p-value** |
|---|---|---|---|
| **Strongly agree** | 20 (47) | 28 (56) | 0.180 |
| **Agree** | 18 (42) | 17 (34) | |
| **Not sure** | 4 (9) | 2 (4) | |
| **Disagree** | 1 (2) | 1 (2) | |
| **Strongly disagree** | 0 (0) | 2 (4) | |

**Supplementary Material Table 5b.** Participant agreement with the statement “The side effects of the pain killers were minimal.”
